# Characterization of Quadratic Nonlinearity between Motion Artifact and Acceleration Data and its Application to Heartbeat Rate Estimation

**DOI:** 10.3390/s17081872

**Published:** 2017-08-14

**Authors:** Sunho Kim, Sungbin Im, Taehyung Park

**Affiliations:** 1 School of Electronic Engineering, Soongsil University, Seoul 06978, Korea; serika@ssu.ac.kr; 2 Department of Industrial and Information Systems Engineering, Soongsil University, Seoul 06978, Korea; tpark@ssu.ac.kr

**Keywords:** motion artifact, accelerometer, nonlinear modeling, cross bicoherence test, Volterra filter, photoplethysmography, heartbeat rate monitoring

## Abstract

Accelerometers are applied to various applications to collect information about movements of other sensors deployed at diverse fields ranging from underwater area to human body. In this study, we try to characterize the nonlinear relationship between motion artifact and acceleration data. The cross bicoherence test and the Volterra filter are used as the approaches to detection and modeling. We use the cross bicoherence test to directly detect in the frequency domain and we indirectly identify the nonlinear relationship by improving the performance of eliminating motion artifact in heartbeat rate estimation using a nonlinear filter, the second-order Volterra filter. In the experiments, significant bicoherence values are observed through the cross bicoherence test between the photoplethysmogram (PPG) signal contaminated with motion artifact and the acceleration sensor data. It is observed that for each dataset, the heartbeat rate estimation based on the Volterra filter is superior to that of the linear filter in terms of average absolute error. Furthermore, the leave one out cross-validation (LOOCV) is employed to develop an optimal structure of the Volterra filter for the total datasets. Due to lack of data, the developed Volterra filter does not demonstrate significant difference from the optimal linear filter in terms of t-test. Through this study, it can be concluded that motion artifact may have a quadaratical relationship with acceleration data in terms of bicoherence and more experimental data are required for developing a robust and efficient model for the relationship.

## 1. Introduction

Recently, according to development of wearable device technologies, the usage of accelerometers has been increased to collect information about movements of sensors deployed at various body parts and human motion [1,2]. To obtain the accurate information from various kinds of sensors, especially, studies are actively performed for minimizing the influence of the motion artifact using the acceleration data of the acceleration sensor.

A representative application is the estimation of heartbeat rate during exercise using photoplethysmography (PPG) signal. This measurement by a wearable device is carried out through the real-time estimation of heartbeat rate by acquiring a PPG signal from the wrist area, instead of using an electrocardiogram (ECG) signal [3,4,5]. However, there are several obstacles to properly measuring heartbeat rate using a PPG signal measured at the wrist. Note that exercise beyond a certain level distorts the PPG signal at the time of signal acquisition. The motion artifact usually presents notable energy, as it possesses a similar frequency to that of the heartbeat. Therefore, many techniques ranging from the conventional filter to the compressed sensing are investigated to estimate an accurate heartbeat rate by overcoming the influence of motion artifacts.

First, according to [6], an adaptive noise cancellation (ANC) method demonstrates extreme performance difference depending on the use of measured or synthesized accelerometer signals. In the case of using synthesized accelerometer signals, ANC is very vulnerable to the motion artifacts while ANC with measured accelerometer signals shows fairly good performance for mild motion artifacts [7,8,9,10,11].

The approach based on the wavelet transform [12,13] has a drawback that its performance is dependent on the threshold value that should be chosen because the reconstructed signal waveform relies on this value of threshold which may not be optimal in all users. Therefore, further studies need to be done on selecting the optimal threshold value for better and more robust performance [14,15].

Another method is presented using the Kalman filter [16,17]. The Kalman filter approach has a difficulty of finding suitable initial values for its filter coefficients since the system model in the Kalman filter requires information on certain targets, including the motion artifact variance and PPG signal’s characteristics. It is known that the estimation performance is similar to that of ANC [14].

For the successful application of independent component analysis (ICA), some factors should be carefully considered [18]. For the ICA model, the number of independent sources can affect the process of the pursuit of statistical independence from multivariate statistical data. This approach basically requires the assumption that heartbeat rate and motion artifact signals are independent of one another. However, there is actual overlapping of the frequency range, even if there is an increase in independency to a certain degree due to the preprocess procedure, and its performance degrades significantly [14,19]. For mitigating this degradation, the ICA approach, presented by Kim and Yoo, is to employ preprocessing such as block interleaving [20].

It is known that the SVD-based schemes are robust to motion artifacts compared to other approaches. Unfortunately, it goes through performance degradation to aperiodic and/or intermittent motion artifacts such as warming-up before exercise [21].

Recently, the approach, which is based on signal decomposition [22,23,24], demonstrates the best performance among the existing approaches but because its computational load is too heavy, it cannot be applied to the wearable devices requiring real-time processing. Furthermore, the approach presented in [24] employs the second-order Volterra filter to nonlinearly model motion artifacts with signal decomposition. This shows the possibility on the nonlinear relationship between the motion artifact interference and the acceleration data.

In this study, the relationship between the motion artifact and the acceleration data is investigated in terms of nonlinearity. Since it is impossible to measure pure motion artifact signals, in this study, the measured PPG signal with motion artifact is employed under the assumption that they are uncorrelated to each other. The cross bicoherence test [25,26,27] is employed to detect the quadratic nonlinearity in the relationship. The cross bicoherence test is a well-known statistical approach to detection of quadratic nonlinearity involved in various phenomena.

The following approach is the application of the second-order Volterra filter to the heartbeat rate estimation. The Volterra filter is applied to quadratically model the relationship using the measured PPG signal with motion artifact, and the acceleration data samples and then the measured PPG signal is subtracted by its filter output, the motion artifact estimate, to obtain the residue signal. This residual signal is used to estimate the heartbeat rate. In this experiment, compared to the performance of the linear filter based approach, the improvement obtained by the second-order Volterra filter is investigated to confirm the quadratic relationship. Furthermore, the validity of this approach is tested with the leave one out cross validation (LOOCV) [28].

This paper is organized as follows. In Section 2, the source and the data collection conditions of the PPG datasets used in this study are presented and the performance measure is introduced for the heartbeat rate estimation. In Section 3, the signal model considered in the study and the related assumptions are explained. In Section 4, the cross bicoherence test and the second-order Volterra filter are introduced as the approaches to detection and quantification and the framework of the heartbeat rate estimation with the PPG signals is briefly described. Section 5 presents the cross bicoherence test results, the heartbeat rate estimation results with the linear and Volterra filters, the application of LOOCV and the discussion. Finally, the paper is concluded in Section 6.

## 2. Datasets and Performance Measure

The database that was provided for 2015 IEEE Signal Processing Cup [29] is employed in this study. The database consists of twelve datasets of measurements. Each dataset contains the signal samples composed of two PPG signals, the acceleration data of three axes, and the ground-truth heartbeat rates in BPM. The sampling rate of signals is 125 Hz. Further information on the data conditions are presented in [22,24,30,31].

The performance of heartbeat rate estimation is evaluated in terms of average absolute error (AAE) over the total dataset, which is defined as

(1) $$\mu =\frac{1}{N}\sum_{i=1}^{N}|BPM_{est}\left(i\right)-BPM_{true}\left(i\right)|,$$

where *N* denotes the total number of time windows, and *i* represents the window number. $BPM_{est}\left(i\right)$ denotes the estimated heartbeat rate, while $BPM_{true}\left(i\right)$ is the corresponding ground-truth of the heartbeat rate, which was provided with the datasets.

## 3. Signal Model

It is assumed in this study that the measured PPG signal p(n) is composed of three components [32,33,34,35]:

(2) p(n)=b(n)+m(n)+v(n)

where b(n) represents the waveform that contains the information on heartbeat to be measured. Signal m(n) refers to the interference signal introduced by motion artifacts to the measured PPG signal, while v(n) is zero-mean additive white Gaussian noise (AWGN), which is statistically independent of b(n) and m(n). It is also assumed in this study that the waveform b(n) is independent of the interference m(n) [20,32,33]. Note here that the interference signal m(n) will be nonlinearly modeled based on $a_{x}\left(n\right)$, $a_{y}\left(n\right)$, and $a_{z}\left(n\right)$ that represent the acceleration data of the *x*-, *y*-, and *z*-axes, respectively.

The existing approaches to mitigating motion artifact interference using acceleration data are mainly based on the assumption on the linear relationship between the interference and the acceleration data [32,33,34]. However, according to [36], the mean power due to the rate of change of kinetic energy generated by translation motion is proportional to the integral of squared acceleration output. Thus, the interference signal power due to motion artifact is supposed to be a function of the squared acceleration data. The focus here is on the detection of the nonlinear relationship between motion artifact interference and acceleration data. In the first stage, the application of the cross bicoherence test is considered since the bicoherence test is a powerful statistical concept for analyzing data associated various nonlinear phenomena [25,26,27].

## 4. Approaches

### 4.1. Cross Bicoherence Test

In order to detect and quantify the nonlinear interaction between motion artifact interference and acceleration data of each axis, the cross bicoherence test is applied to the datasets.

Consider two fluctuations x(n) and y(n) and their Fourier transform pair X(f) and Y(f), respectively. The cross bispectrum $S_{YXX}\left(f_{1},f_{2}\right)=E\left\{Y\left(f_{1}+f_{2}\right)X^{*}\left(f_{1}\right)X^{*}\left(f_{2}\right)\right\}$ is a triple correlation function that measures the correlation between $Y(f_{1}+f_{2})$ and $X(f_{1})$ and $X(f_{2})$ at the frequency $\left(f_{1},f_{2},f_{1}+f_{2}\right)$ [26]. A normalized version of degree of this nonlinear correlation is given by the cross bicoherence spectrum,

(3) $$b_{c}^{2}\left(f_{1},f_{2}\right)=\frac{|S_{YXX}\left(f_{1},f_{2}\right){|}^{2}}{E\{|Y\left(f_{1}+f_{2}\right){|}^{2}\}E\{|X\left(f_{1}\right)X\left(f_{2}\right){|}^{2}\}},$$

which is bounded by zero and unity, that is,

(4) $$0\leq b_{c}^{2}\left(f_{1},f_{2}\right)\leq 1.$$

Since the cross bicoherence $b_{c}\left(f_{1},f_{2}\right)$ is normalized coherence function, it is very useful for detecting and quantifying the quadratically nonlinear relationship between two fluctuations x(n) and y(n). Since the cross bicoherence is to be implemented digitally, note that $f_{1}+f_{2}\leq f_{s}/2$ and $f_{2}\leq f_{s}/2$, where $f_{s}$ represents a sampling rate.

In this study, the motion artifact interference m(n) is not directly measurable but the motion artifact interference is included in the measured PPG signal p(n) as mentioned in Equation (2). Thus, the measured PPG signal is employed for the cross-bicoherence test as Y(f) and the acceleration data of each axis are used as X(f) in Equation (3). That is, Equation (3) can be rewritten as follows:

(5) $$b_{c,\kappa }^{2}\left(f_{1},f_{2}\right)=\frac{|S_{MA_{\kappa }A_{\kappa }}\left(f_{1},f_{2}\right){|}^{2}}{E\{|P\left(f_{1}+f_{2}\right){|}^{2}\}E\{|A_{\kappa }\left(f_{1}\right)A_{\kappa }\left(f_{2}\right){|}^{2}\}}\;\left(\kappa \in \left\{x,y,z\right\}\right),$$

where P(f) is the discrete Fourier transform of p(n) and $A_{\kappa }$ with κ∈{x,y,z} represent the discrete Fourier transform of the acceleration data $a_{\kappa }\left(n\right)$ of *x*-, *y*-, and *z*-axes, respectively. Note that the motion artifact interference is scalar and may have relationship with one , two, or all of the three axis components. Therefore, the cross bicoherence spectrum $b_{c,\kappa }^{2}\left(f_{1},f_{2}\right)$ with κ∈{x,y,z} should be investigated for all combinations of frequency pairs. Experimental determination of nonzero cross bicoherence represents the existence of quadratically nonlinear relationship between the motion artifact interference and the acceleration data of each axis.

After detecting the nonlinearity in the relationship between the motion artifact interference and the acceleration data of each axis, to characterize the quadratically nonlinear relationship, the second-order Volterra filter is considered in this study. The Volterra filter can be regarded as a generalized form of the Taylor series with memory [37]. When modeling a nonlinear input-output relationship, the Volterra filter is widely applied because it is a kind of black-box modeling approaches, which requires no information on the internal structure of an unknown system.

### 4.2. A Second-Order Volterra Filter

In this study, it is assumed that the acceleration data of three axes are regarded as inputs to an unknown nonlinear system, which produces the motion artifact interference signal as a corresponding output. Under this assumption, the second-order multichannel Volterra filter is considered for modeling the unknown system because acceleration data of three axes are input to the unknown system. In addition, one of the disadvantages of the Volterra model is that it consists of many model coefficients, which requires a large size data to estimate its coefficients. Since the number of data samples in this study is limited, it is required to reduce the number of coefficients, therefore the application of a sparse model that utilizes the coefficients selected based on various criteria including magnitude, memory span, contribution, and so on.

The model used in the study is the sparse second-order Volterra filter, given by

(6) $$\begin{array}{c} y\left(n\right)=y_{l}\left(n\right)+y_{q\kappa }\left(n\right),\;\;\left(\kappa \in \left\{x,y,z\right\}\right), \end{array}$$

where

(7) $$\begin{array}{cc} y_{l}\left(n\right) & =\sum_{k=0}^{N_{l}-1}h_{lx}\left(k\right)a_{x}\left(n-k\right)+\sum_{k=0}^{N_{l}-1}h_{ly}\left(k\right)a_{y}\left(n-k\right)+\sum_{k=0}^{N_{l}-1}h_{lz}\left(k\right)a_{z}\left(n-k\right) \end{array}$$

and

(8) $$\begin{array}{cc} y_{q\kappa }\left(n\right) & =\sum_{q_{1}=0}^{N_{q}-1}\sum_{q_{2}=q_{1}}^{N_{q}-1}h_{q\kappa }\left(q_{1},q_{2}\right)a_{\kappa }\left(n-q_{1}\right)a_{\kappa }\left(n-q_{2}\right). \end{array}$$

In Equation (7), $h_{lx}$, $h_{ly}$, and $h_{lz}$ represent the linear coefficients for the three axes, respectively, while $h_{q\kappa }$ in Equation (8) does the quadratic coefficients for one of the three axes, that is , one of $h_{qx}$, $h_{qy}$, and $h_{qz}$. Thus, the model in Equation (6) consists of the linear components of the tri-axis acceleration data and the quadratic component of single-axis acceleration data among the three axes. It is known that the quadratic kernels are symmetric since $h_{q\kappa }\left(q_{1},q_{2}\right)$ and $h_{q\kappa }\left(q_{2},q_{1}\right)$ cannot be distinguished from each other. For this reason the double summation in Equation (8) is carried from $q_{2}=q_{1}$. The complete version of the model in Equation (6) requires $3N_{l}+N_{q}\left(N_{q}+1\right)/2$ coefficients, which implies more data for robust estimation.

In order to estimate the coefficients in Equations (7) and (8) for modeling the nonlinear system that relates three axes input data to the motion artifact interference, the Wiener-Hopf equation approach is applied to the dataset [38] as follows:

(9) $$h^{T}=\left(yX^{T}\right){\left(XX^{T}\right)}^{-1}.$$

where the vector h represents the $\left(3N_{l}+N_{q}\left(N_{q}+1\right)/2\right)$-by-1 optimum coefficient vector of the Volterra model of Equation (6), that is,

(10) $$h={\left[h_{lx}\;h_{ly}\;h_{lz}\;h_{q\kappa }\right]}^{T}$$

where $h_{l\kappa }={\left[h_{l\kappa }\left(0\right),\ldots h_{l\kappa }\left(N_{l}-1\right)\right]}^{T}$ and $h_{q\kappa }=[h_{q\kappa }\left(0,0\right),h_{q\kappa }\left(0,1\right),\ldots ,h_{q\kappa }\left(0,N_{q}-1\right),h_{q\kappa }\left(1,1\right),\ldots ,$ $h_{q\kappa }\left(N_{q}-1,N_{q}-1\right){]}^{T}$ with κ∈{x,y,z}. The input matrix X contains the input vectors $x={\left[x_{lx},x_{ly},x_{lz},x_{q\kappa }\right]}^{T}$ corresponding to h, thus $XX^{T}$ represents the auto-correlation for the input vector while the vector $yX^{T}$ does the cross-correlation between the acceleration data vector and the measured PPG signal vector.

### 4.3. Heartbeat Rate Estimation Using PPG Signals

**Figure 1:**
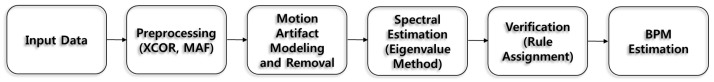
Functional block diagram of the heartbeat rate estimation.

In this subsection, the pre-processing and the post-processing required for heartbeat rate estimation are presented. For performance comparison with the linear modeling results, the heartbeat rate estimation process employed in this study is very similar to the process presented in the reference [33]. Figure 1 displays the functional block diagram of the scheme considered in this study.

The estimation procedure uses two PPG signals, which are simultaneously measured by two pulse oximeters with green LEDs that are 2 cm apart, denoted by $p_{1}\left(n\right)$ and $p_{2}\left(n\right)$. Due to the characteristics of PPG sensors, measuring signals is highly dependent on the wearer’s motion or location and the input signal may have a small delay with respect to each other due to mechanical characteristics of the sensor. This phenomenon can be avoided by using more than one sensor. Each PPG signal is filtered by the moving average filter to reduce the noise and fluctuation of the signal. Based on the observation on the performances with various filter orders, the filter order is selected as seven.

In this study, assuming that the acceleration data is correlated with the measured PPG signal, in Equation (2), the acceleration signal is applied to model the interference signal m(n) resulting from the motion artifact. The modeling is carried out by finding the coefficient vector h using Equation (9). Then, the estimated interference signal $\hat{m}=hX$ is removed from the measured PPG signal. The residual signal, $y_{\mathrm{res}}=y-\hat{m}$, theoretically contains only the signal related to heartbeat rate information.

In the block denoted by Spectral Estimation in Figure 1, the spectral estimation based on the eigenvalue method is applied to the residual signal $y_{\mathrm{res}}$ to obtain the spectral component corresponding to heartbeat rate [38,39]. The eigenvector method, one of the noise subspace methods for frequency estimation, is very similar to the multiple signal classification algorithm, but its features produce fewer spurious peaks and more accurately estimate frequency components for the signals of line spectra. Thus, the pseudospectrum can be effectively utilized for estimating the frequency component corresponding to heartbeat rate [40,41,42]. For efficient and accurate heartbeat rate estimation from the estimated spectrum, the frequency search range is confined to [0.86 Hz, 3.03 Hz], which corresponds to 51.6 BPM through 181.8 BPM. This falls into the range of observable heartbeat rates considering the traits of human bodies.

The verification stage in Figure 1 selects the frequency component that has the peak value under several constraints on the spectrum. As mentioned above, since the experiment is performed on the two PPG signals $p_{1}\left(n\right)$ and $p_{2}\left(n\right)$, respectively, two estimated BPM values are available with respect to each time window. In the first window, the estimated BPM is set to the mean value of the two BPM estimated from the PPG signals $p_{1}\left(n\right)$ and $p_{2}\left(n\right)$, respectively. In the window with window number i≥2, among the two estimated BPM values, as $BPM_{est}\left(i\right)$, the algorithm chooses the value that is the closest to the estimated BPM decided in the previous time window of window number i−1.

In practical applications, a dramatic change in the estimation value occurs with very severe motion artifacts or with the insertion of a particular type of motion artifacts [22,33]. An abrupt change in heartbeat rate is very rare to observe in actual biological change. For mitigating this malfunction, several simple rules are applied in this study, which were developed for the approach with the linear filters [33]. It is clear that a change in heartbeat rate follows certain trends, under the assumption that changes in the heartbeat rate of human bodies are attributable to exercise or to a biological change. In other words, the actual heartbeat rate increases or decreases at certain cycles in accordance with biological activities. Although the actual heartbeat rate can be increased in a short period of time with strenuous exercise, this does not present a dramatic decrease compared to the increase, even when rest is taken afterwards. Considering this fact, heartbeat rate estimation is conducted by adding 1.75 BPM to the previous estimated value with an increase of U=25 BPM or higher and by subtracting 1.5 BPM from the previous estimated value with a decrease of D=16 BPM or higher. That is,

(11) $$\begin{array}{c} BPM_{est}\left(i\right)=BPM_{est}\left(i-1\right)+1.75,\;\;\mathrm{when}\;\;BPM_{est}\left(i\right)-BPM_{est}\left(i-1\right)>25\\ BPM_{est}\left(i\right)=BPM_{est}\left(i-1\right)-1.5,\;\;\mathrm{when}\;\;BPM_{est}\left(i\right)-BPM_{est}\left(i-1\right)<-16 \end{array}$$

where $BPM_{est}\left(i\right)$ represents the estimated BPM values at time window *i*.

## 5. Results

### 5.1. Application of Cross Bicoherence Test

This subsection presents the results obtained by applying the cross bicoherence test described in Section 4.1 to the datasets. In the experiments, the two signals PPG1 and PPG2 of each data set are filtered with the bandpass filter, which has the passband from 0.5 Hz to 3 Hz. The frequency components in the passband correspond to the heartbeat rates from 30 BPM to 180 BPM, that is the range of interest in this study. Since each data set has two PPG signals and three acceleration data, 72 tests are carried out for the twelve datasets. In the test, the bicoherence is measured using 1024-point FFT, 75% overlap for 1000 samples per segment, and Hanning windowing.

**Figure 2:**
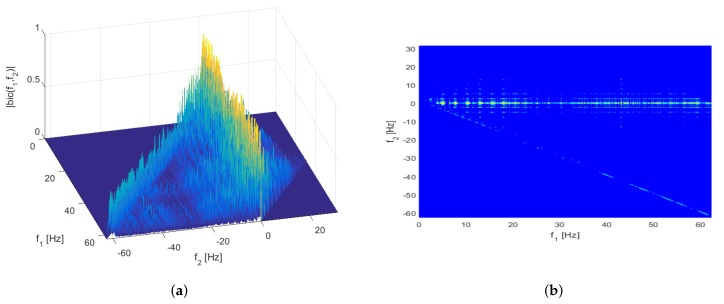
Mesh (**a**) and contour (**b**) plots of the cross bicoherence between PPG1 signal and *y*-axis acceleration data for dataset 10.

**Figure 3:**
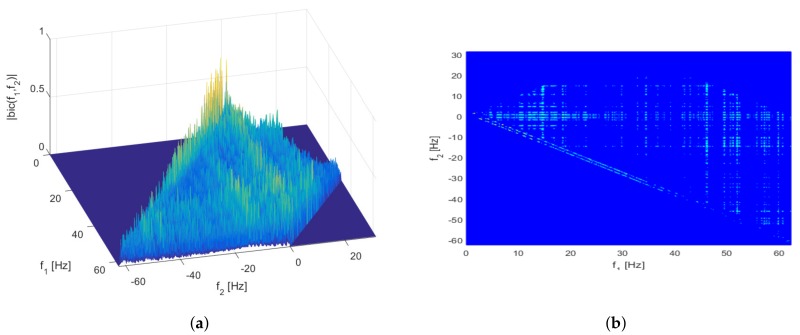
Mesh (**a**) and contour (**b**) plots of the cross bicoherence between PPG1 signal and *z*-axis acceleration data for dataset 2.

Two representative test results from the overall ones are shown in Figure 2 and Figure 3. The graphs shown in the figures are a mesh plot of the magnitude of the cross bicoherence $\left|bic(f_{1},f_{2})\right|$ and its corresponding contour plot. The *x*- and *y*-axes in the graph denote the frequency pair $f_{1}$ and $f_{2}$, respectively.

Figure 2 is the result of the bicoherence estimation between the PPG1 signal and the *y*-axis acceleration data of the dataset 10. This achieves the maximum bicoherence compared to other estimation results. The maximum bicoherence is 0.9346 at the input frequency pair $f_{1}$ = 12.9395 Hz and $f_{2}$ = 0.1221 Hz. Note that this pair corresponds to the output frequency $f_{1}+f_{2}$ = 13.0615 Hz, which is equivalent to 783.69 bpm. Since the frequency with the maximum bicoherence is outside the band of interest, it does not actually affect the heartbeat rate estimation.

On the other hand, Figure 3 shows the maximum bicoherence in the range that affects heartbeat rate measurement. Figure 3 shows the bicoherence test between the *z*-axis acceleration data and the PPG1 signal for dataset 2. The peak coherence is about 0.7130 at $f_{1}$ = 2.8076 Hz and $f_{2}$ = −1.3428 Hz, ($f_{1}+f_{2}$ = 1.4648 Hz), that is, 87.89 bpm.

The maximum values of the cross bicoherence measured between two PPG signals and tri-axis acceleration data in each dataset are listed in Table 1 and Table 2, respectively. For the PPG1 signal, the average of the maximum bicoherence values is 0.6672 and its standard deviation is 0.1373 while the average is 0.6964 and the standard deviation is 0.1187 for the PPG2 signal.

Note that this experiment measures the cross bicoherence between PPG signals, which are contaminated with motion artifact, and tri-axis acceleration data. Therefore, even though a large bicoherence implies the nonlinear relationship, it is difficult to judge whether this nonlinear relationship is related to pure PPG signal or to motion artifact. In order to clear up this ambiguity, this is verified by performing the experiment to be presented in the next section.

### 5.2. Application to Heartbeat Rate Estimation

In order to investigate the quadratic nonlinear relationship between motion artifact interference and acceleration data, in this section the degree of performance improvement is evaluated by estimating the heartbeat rate using the linear filter and the second-order Volterra filter mentioned in Section 4.2. The concrete approach is to estimate the heartbeat rate through the PPG signal in which the motion artifact is modeled and removed by the filters and to compare the estimate with the ground-truth value to measure the AAE.

In this experiment, the time window size for estimating heartbeat rate is an eight-second period. The estimation is performed by overlapping windows with sliding a two-second period. That is, each window is composed of 1000 samples and the number of the overlapped samples in each window is 750 samples.

**Figure 4:**
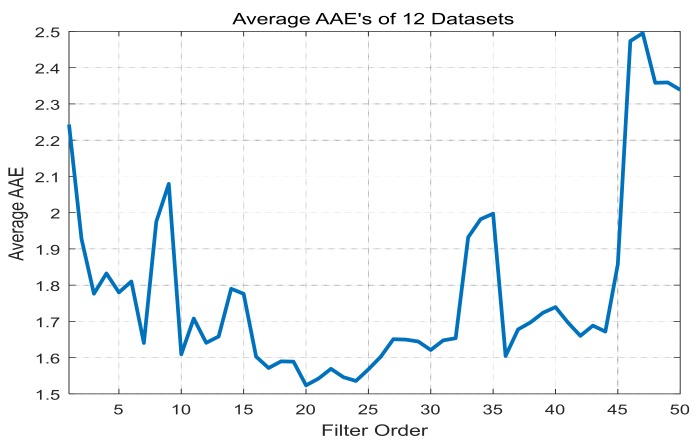
Average values of AAE’s using the linear filter with respect to various filter orders for 12 total datasets.

First, the order of the linear filter is increased from 1 to 50 for 12 data sets using the linear filter given in Equation (7), and the change of AAE is examined. For each filter order, the average value of AAE’s over the total data sets is plotted in Figure 4. As shown in the figure, as the filter order increases, the average AAE tends to decrease until the minimum value, while it tends to increase for the filter orders greater than the filter order that can obtain the minimum value. This implies that the added filter coefficients are not efficient for modeling the motion artifact. Table 3 summarizes the minimum value of AAE and its corresponding linear filter order for each dataset.

The next step is to evaluate the performance of the approach with a second-order Volterra filter given in Equation (6) for the datasets. Note that the second-order Volterra filter considered in this experiment consists of the linear filter, which is same to the previous case, and the quadratic filter described in Equation (8). In the experiment, the parameter κ in Equation (8) is changed to *x*, *y* and *z*, for each axis acceleration data, and the linear filter order $N_{l}$ and the quadratic filter order $N_{q}$ are increased from 1 to 30 to model the motion artifact. The procedure of determining the heartbeat rate presented in Section 4.3 is applied to obtain the heartbeat rate estimate and AAE is measured with respect to the ground-truth of heartbeat rate. The results are summarized in Table 4 according to axis, linear filter order, quadratic filter order and AAE. Compared with those of Table 3 in terms of AAE, except datasets 5 and 7, all the second-order Volterra filters achieve better AAE’s than the linear filters. It is also observed in Table 4 that the linear filter orders $N_{l}$ of the second-order Volterra filters are smaller than those of the linear filters in Table 3 except for dataset 11.

### 5.3. Model Cross Validation

In the previous subsection, the nonlinear relationship between the motion artifact and the acceleration data is investigated through applying the second-order Volterra filter for each dataset. It is observed that the use of the Volterra filter makes it possible to estimate the heartbeat rate relatively accurately in terms of AAE. Unfortunately, note that the twelve datasets are not sufficient for claiming the superiority of linear or Volterra filters in this experiment. Therefore, in order to mitigate this problem due to lack of data, the leave one out cross validation (LOOCV) technique is employed to identify the average AAE values for the entire datasets and to perform the *t*-test with *p*-value to verify the statistical reliability [28].

For the LOOCV approach, the total twelve datasets are divided into one training dataset and eleven test datasets. Each training dataset is selected sequentially from the total datasets and then the remaining datasets become the test datasets.

**Figure 5:**
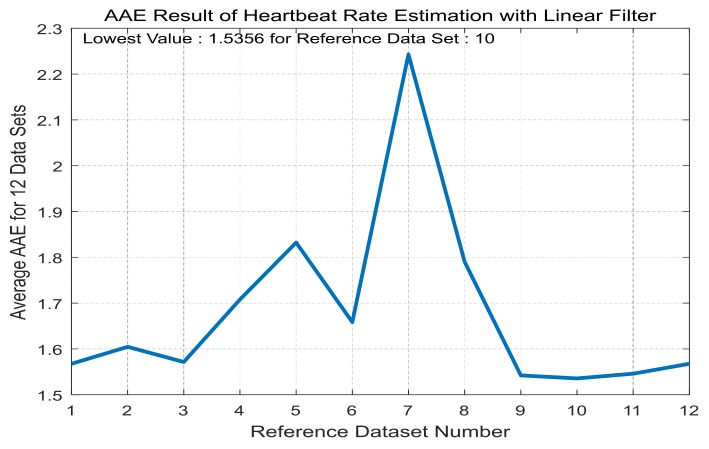
Average AAE’s of the linear filters in Table 3 over the total datasets for LOOCV application.

**Figure 6:**
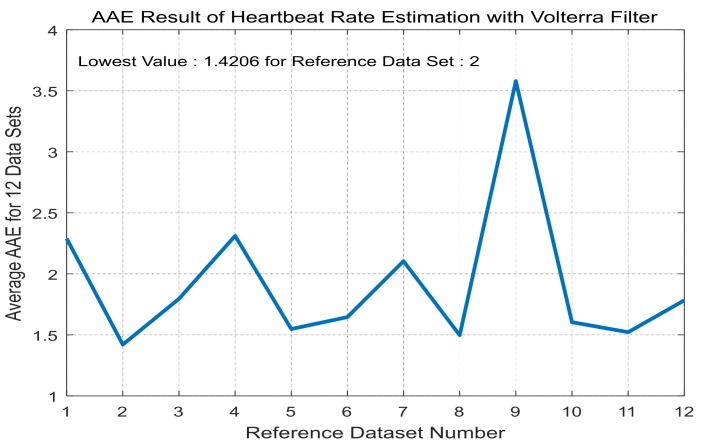
Average AAE’s of the Volterra filters in Table 4 over the total datasets for LOOCV application.

**Figure 7:**
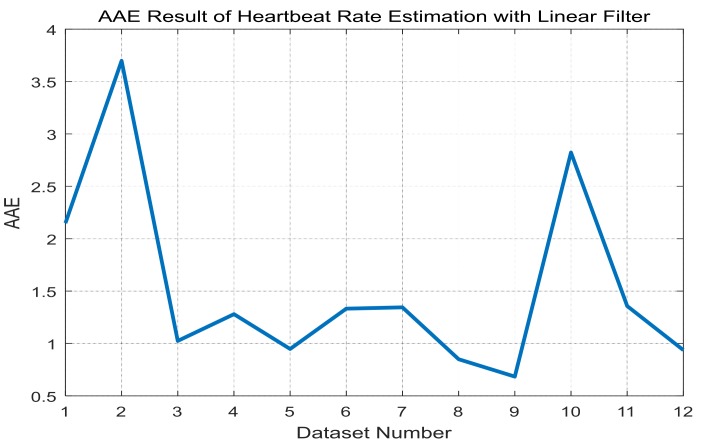
AAE’s of the linear filter of dataset 10 for each dataset.

**Figure 8:**
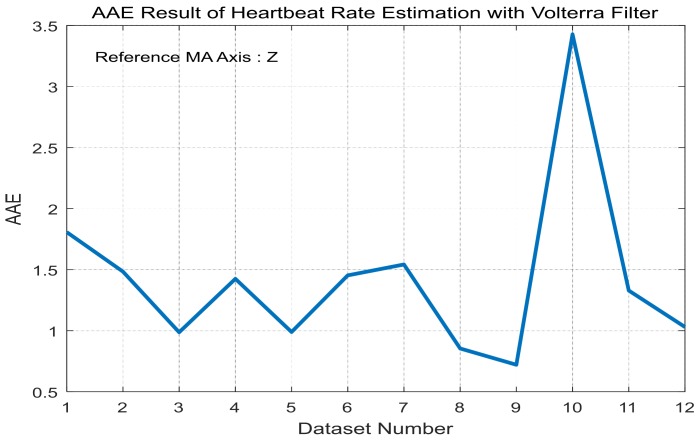
AAE’s of the Volterra filter of dataset 2 for each dataset.

For each training dataset, the optimal linear and Volterra filters that achieve minimum AAE can be found in Table 3 and Table 4, respectively. They are applied to the test datasets to obtain average AAE’s over the total datasets, which are shown in Figure 5 and Figure 6, respectively, for the linear and Volterra filters. Based on the observations on Figure 5 and Figure 6, the linear filter for dataset 10 is selected while the Volterra filter for dataset 2 is done. Mean and standard deviation of the linear filter’s AAE are 1.5356 and 0.9068, respectively, while those of the Volterra filter’s AAE are 1.4206 and 0.7099. AAE’s obtained by the two filters are plotted in Figure 7 and Figure 8 for each dataset. In order to evaluate the significant difference for the two filters, the *t*-test is applied to AAE’s in the figures to achieve *p*-value of 0.7329. This indicates that the there is no significant difference between the two filter’s AAE performance for these datasets.

## 6. Conclusions

As the application fields of various sensors are diversified, accurate measurement using sensors becomes an important issue. In the environment where motion artifact exists, especially, there is a problem that the measurement through sensors becomes inaccurate. As a solution to these problems, researches are being actively carried out to eliminate the motion artifact by using the acceleration sensor. In this study, the cross bicoherence is measured and the Volterra filter is applied to modeling the relationship between the motion artifact and the acceleration data under the hypothesis that the relationship is nonlinear, which is different from the previous studies that assume a linear relationship. The cross-bicoherence test results show that there is a significant nonlinear relationship between the motion artifact and the acceleration data. Based on the results, it is expected that more accurate measurement can be made by removing motion artifact by performing nonlinear modeling between motion artifact and acceleration data. Unfortunately, due to lack of data, an optimal structure of Volterra filter, which outperforms a linear filter, cannot be developed in this work. For further research, a more compact and computationally efficient model needs to be investigated for various applications since the Volterra filter employed in this work generally requires many filter coefficients to describe nonlinear relationship, which is a hurdle to many applications. 

## Figures and Tables

**Table 1:** Maximum values of the cross bicoherence of the PPG1 signal with respect to tri-axis acceleration data.

Dataset	*x*-axis				*y*-axis				*z*-axis		
	$f_{1}$ (Hz)	$f_{2}$ (Hz)	Coherence		$f_{1}$ (Hz)	$f_{2}$ (Hz)	Coherence		$f_{1}$ (Hz)	$f_{2}$ (Hz)	Coherence
data 1	5.859	−2.808	0.562		4.395	−1.465	0.484		8.789	−5.859	0.556
data 2	2.808	−1.343	0.731		6.958	−5.493	0.722		2.808	−1.343	0.713
data 3	2.808	−1.343	0.577		2.808	−1.343	0.603		8.545	−7.080	0.578
data 4	4.639	−2.686	0.359		43.457	−41.138	0.396		43.579	0.000	0.854
data 5	49.927	0.610	0.687		5.615	−4.150	0.675		61.401	0.000	0.742
data 6	5.005	−3.784	0.654		5.005	−3.784	0.618		8.789	−7.568	0.613
data 7	5.127	−3.784	0.526		2.686	−1.343	0.485		2.564	−1.221	0.502
data 8	11.719	−8.789	0.720		60.913	0.000	0.857		7.324	−4.395	0.724
data 9	5.981	−4.883	0.574		2.319	−1.221	0.677		57.739	0.000	0.757
data 10	3.662	0.122	0.766		12.939	0.122	0.935		6.470	−0.366	0.912
data 11	4.517	−2.930	0.697		58.838	−0.122	0.919		7.446	−5.859	0.660
data12	4.517	−1.465	0.722		5.981	−2.930	0.697		59.204	0.000	0.766

**Table 2:** Maximum values of the cross bicoherence of the PPG2 signal with respect to tri-axis acceleration data.

Dataset	*x*-axis				*y*-axis				*z*-axis		
	$f_{1}$ (Hz)	$f_{2}$ (Hz)	Coherence		$f_{1}$ (Hz)	$f_{2}$ (Hz)	Coherence		$f_{1}$ (Hz)	$f_{2}$ (Hz)	Coherence
data1	3.052	−1.587	0.568		4.517	−3.052	0.521		7.446	−5.981	0.595
data2	1.465	1.343	0.657		57.495	0.000	0.698		6.958	−4.150	0.641
data3	3.784	−2.563	0.506		2.441	−1.221	0.481		7.690	−6.470	0.507
data4	5.737	−4.272	0.631		4.273	−2.808	0.642		43.579	0.000	0.854
data5	49.927	−0.610	0.688		2.808	−1.343	0.664		61.401	0.000	0.743
data6	2.441	−1.221	0.682		2.441	−1.221	0.695		2.441	−1.221	0.698
data7	5.127	−3.784	0.742		2.686	−1.343	0.712		2.563	−1.221	0.728
data8	1.587	1.465	0.722		60.913	0.000	0.857		1.587	1.465	0.724
data9	7.202	−6.104	0.517		2.319	−1.221	0.610		57.739	0.000	0.757
data10	3.662	0.122	0.795		12.940	−0.122	0.935		3.784	0.366	0.915
data11	3.052	−1.465	0.631		58.838	0.122	0.919		7.446	−5.859	0.643
data12	2.930	−1.343	0.799		3.052	−1.465	0.795		4.395	−2.808	0.803

**Table 3:** AAE values obtained by performing motion artifact removal using the linear filter.

Dataset	1	2	3	4	5	6	7	8	9	10	11	12
$N_{l}$	25	36	17	11	4	13	1	14	21	24	23	25
AAE	2.01	1.59	0.97	1.14	0.82	1.16	0.80	0.80	0.66	2.82	1.23	0.92

**Table 4:** AAE values obtained by performing motion artifact removal using the second-order Volterra filter.

Dataset	1	2	3	4	5	6	7	8	9	10	11	12
Axis	*y*	*z*	*z*	*y*	*z*	*y*	*x*	*y*	*y*	*y*	*x*	*z*
$N_{l}$	17	29	14	9	3	10	1	10	2	5	25	13
$N_{q}$	24	4	9	6	3	2	3	11	8	19	7	10
AAE	1.61	1.48	0.93	1.10	0.83	1.08	0.84	0.74	0.63	2.19	1.16	0.79
